# 1,25D_3_ differentially suppresses bladder cancer cell migration and invasion through the induction of miR-101-3p

**DOI:** 10.18632/oncotarget.19629

**Published:** 2017-07-27

**Authors:** Yingyu Ma, Wei Luo, Brittany L. Bunch, Rachel N. Pratt, Donald L. Trump, Candace S. Johnson

**Affiliations:** ^1^ Department of Pharmacology and Therapeutics, Roswell Park Cancer Institute, Buffalo, NY, USA; ^2^ Inova Schar Cancer Institute, Falls Church, VA, USA

**Keywords:** 1,25D_3_, bladder cancer, miR-101-3p, migration, invasion

## Abstract

Metastasis is the major cause of bladder cancer death. 1,25D_3_, the active metabolite of vitamin D, has shown anti-metastasis activity in several cancer model systems. However, the role of 1,25D_3_ in migration and invasion in bladder cancer is unknown. To investigate whether 1,25D_3_ affects migration and invasion, four human bladder cell lines with different reported invasiveness were selected: low-invasive T24 and 253J cells and highly invasive 253J-BV and TCCSUP cells. All of the four bladder cancer cells express endogenous and inducible vitamin D receptor (VDR) as examined by immunoblot analysis. 1,25D_3_ had no effect on the proliferation of bladder cancer cells as assessed by MTT assay. In contrast, 1,25D_3_ suppressed migration and invasion in the more invasive 253J-BV and TCCSUP cells, but not in the low-invasive 253J and T24 cells using “wound” healing, chemotactic migration and Matrigel-based invasion assays. 1,25D_3_ promoted the expression of miR-101-3p and miR-126-3p in 253J-BV cells as examined by qRT-PCR. miR-101-3p inhibitor partially abrogated and pre-miR-101-3p further suppressed the inhibition of 1,25D_3_ on migration and invasion in 253J-BV cells. Further, 1,25D_3_ enhanced VDR recruitment to the promoter region of miR-101-3p using ChIP-qPCR assay. 1,25D_3_ enhanced the promoter activity of miR-101-3p as evaluated by luciferase reporter assay. Taken together, 1,25D_3_ suppresses bladder cancer cell migration and invasion in two invasive/migration competent lines but not in two less invasive/motile lines, which is partially through the induction of miR-101-3p expression at the transcriptional level.

## INTRODUCTION

Bladder cancer is the fourth most commonly diagnosed cancer and the eighth leading cause of cancer death in men [1]. Metastasis is the major cause of bladder cancer related death. The 5-year survival rates for localized and metastatic bladder cancers are 94% and 6%, respectively [2]. Most (75%) patients newly diagnosed with bladder cancer have superficial disease which relatively infrequently becomes invasive and metastatic; by contrast, in most patients who die of metastatic bladder cancer, the tumor is invasive and metastatic at initial diagnosis [3]. Recurrence, low response rate and resistance to chemotherapeutic therapy remain clinical issues. Therefore, new and effective approaches to treat invasive bladder cancer are urgently needed.

1α, 25-dihydroxyvitamin D (1,25D_3_), the active metabolite of vitamin D, may suppress cancer growth and spread by a variety of mechanisms [4-11]. We and others have shown that that 1,25D_3_ potentiates the antitumor activity of the chemotherapeutic agents used most frequently in bladder cancer (cisplatin and gemcitabine) as well as many other classes of antineoplastic drugs; we have shown that this potentiation in human bladder cancer is mediated through the induction of p73 [12]. We also have shown that 1,25D_3_ suppresses migration and invasion of squamous cell carcinoma (SCC) cells, and these effects were accompanied by decreased expression and secretion of MMP-2 and MMP-9 and increased expression of E-cadherin [13]. Other studies show that 1,25D_3_ inhibits prostate cancer cell adhesion, migration and invasion by various mechanisms such as up-regulation of E-cadherin [13], down-regulation of integrins, MMP-9 and cathepsins [14, 15], DICKKOPF-4 [16] or protein kinase A [17]. In contrast to its effect on cancer cells, 1,25D_3_ promotes cell migration of non-cancer vascular smooth muscle cells through PI3K activation [18]. Conversely, vitamin D deficiency promotes metastatic cancer cell growth in several metastasis models [19, 20]. Low serum 25(OH) D_3_ levels at diagnosis are associated with a poorer prognosis in kidney, lung, breast cancer as well as non-Hodgkin lymphoma. These findings are consistent with the hypothesis that 1,25D_3_ suppresses cell growth as well as inhibits cancer cell migration and invasion in multiple cancer types. However, the role of 1,25D_3_ in bladder cancer cell migration and invasion is unclear.

miRNAs have significant and broad effects on tumorigenesis, and cancer progression and metastasis [21-23]. miRNA expression in primary and metastatic tumors may differ substantially, suggesting a role for miRNAs in metastasis [24]. Recent data support a role for miRNAs in bladder cancer progression. Multiple studies analyzed miRNA expression in bladder cancer and show various results [25-30]. miRNAs have been identified as 1,25D_3_ targets [31-33]. 1,25D_3_ induces miR-98 expression which contributes to 1,25D_3_ growth inhibitory effect in prostate cancer cells [31]. 1,25D_3_ induces miR-498 and results in the suppression of human telomerase reverse transcriptase in ovarian cancer cells [34]. 1,25D_3_-induced miR-22 is involved in the anti-proliferative and anti-migratory activities of 1,25D_3_ in colon cancer cells [32]. Vitamin D_3_ supplementation prior to prostatectomy increased tissue and serum levels of 1,25D_3_ in a dose dependent manner and the high dose vitamin D_3_ suppressed PTH and PSA levels [35]. In prostate stroma cells, the expression of miR-126-3p, miR-154-5p and miR-21-5p was positively correlated with 1,25D_3_ prostate tissue content [36]. These data support the role of vitamin D in influencing the biology of tumor tissues. Nevertheless, studies on the regulation of miRNAs by 1,25D_3_ are limited and few studies have reported the effects of 1,25D_3_ on miRNAs in bladder cancer.

In this study, we investigate the effect of 1,25D_3_ on proliferation, migration and invasion in a panel of human bladder cancer cells. In a previous study, we demonstrated that 1,25D_3_ differentially regulates miRNA expression in a pair of human bladder cancer cells, low-tumorigenic and non-invasive 253J and highly-tumorigenic and invasive 253J-BV cells, using miRNA qPCR panels [37]. From the list of differentially regulated miRNAs, we selected miR-101-3p and miR-126-3p based on their potential role in migration and invasion to investigate their role in the regulation of migration and invasion by 1,25D_3_ in bladder cancer cells [38-41]. Further, the potential mechanism for 1,25D_3_ regulation of miRNA expression was studied.

## RESULTS

### VDR is expressed and inducible in human bladder cancer cells

**Figure 1:**
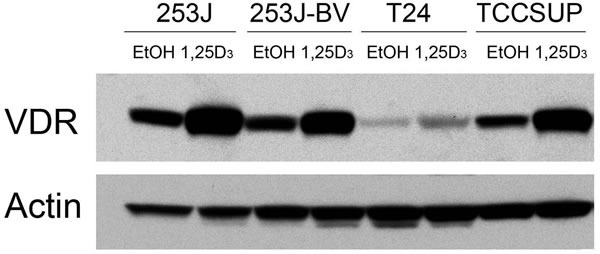
VDR expression in human bladder cancer cells Human bladder cancer cell lines 253J, 253J-BV, T24 and TCCSUP were treated with EtOH or 500 nM 1,25D_3_ for 48 h. VDR protein expression was assessed by immunoblot analysis. Actin was the loading control. Results are representative of two independent experiments.

Four human bladder cell lines with different reported *in vivo* invasiveness were selected: low-invasive T24 and 253J cells and highly invasive 253J-BV and TCCSUP cells [42-44]. In order to initially explore the mechanism whereby these cells might respond to 1,25D_3_, VDR expression was first examined. Although the endogenous levels differ, VDR is expressed and induced by 1,25D_3_ in all four cell lines (Figure 1), indicating that the putative first steps in 1,25D_3_ signaling appears intact in these cell lines.

### 1,25D_3_ does not affect bladder cancer cell proliferation

**Figure 2:**
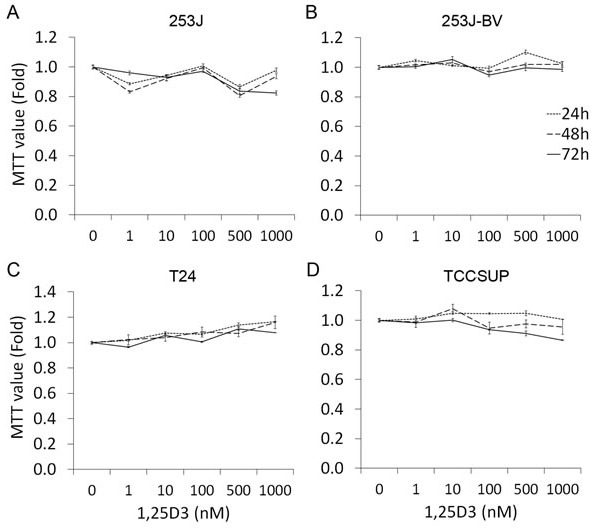
1,25D_3_ has no impact in bladder cancer cell proliferation Human bladder cancer cells were treated with EtOH or 1 - 1000 nM of 1,25D_3_ for 24 to 72 h. Cell proliferation was evaluated by MTT assays. The experiments were run in triplication and the data was presented as the fold of the MTT value of EtOH treatment: **A.** 253J cells, **B.** 253J-BV cells, **C.** T24 cells, and **D.** TCCSUP cells. Results are representative of three independent experiments.

To investigate the impact of 1,25D_3_ in bladder cancer cell proliferation, human bladder cancer cells 253J, 253J-BV, T24 and TCCSUP were treated with varying concentrations (0-1000 nM) of 1,25D_3_ for 24 to 72 h and cell proliferation was assessed by the MTT assay. 1,25D_3_ did not affect the proliferation of the four bladder cancer cell lines (Figure 2).

### 1,25D_3_ regulates bladder cancer cell migration and invasion

**Figure 3:**
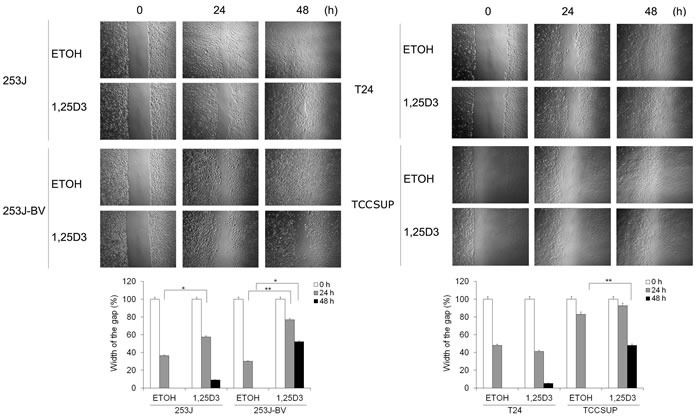
1,25D_3_ differentially inhibits bladder cancer cell migration Wounds were introduced by scratching a monolayer of bladder cancer cells. Cells were treated with EtOH or 500 nM 1,25D_3_. Migration was monitored using a light microscope at 0, 24 and 48 h. The width of the gaps in three experiments was measured and the means and their standard errors (SEM) presented in bar graphs below the images. *, *P* < .05; **, *P* < .01. Results are representative of three independent experiments.

**Figure 4:**
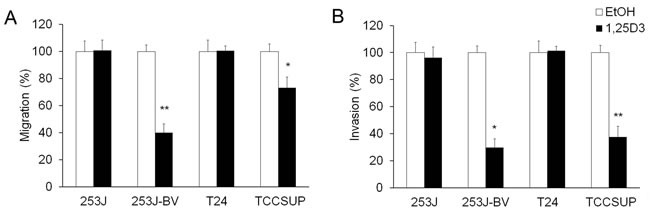
1,25D_3_ differentially regulates migration and invasion of bladder cancer cells Human bladder cancer cell lines were treated with EtOH or 500 nM 1,25D_3_ for 48 h. **A.** Chemotactic migration assays were performed using modified Boyden chamber (8 μm pores) with 5% FBS. **B.** Matrigel-based invasion assays were performed with Boyden chambers with 5% FBS. The cell numbers per field were counted. Migrated or invaded cell numbers relative to EtOH-treated cells were presented in bar graphs. Results are representative of three independent experiments. *, *P* < .05 and **, *P* < .01 in Student's t tests comparing EtOH and 1,25D_3_ treatments.

To investigate the impact of 1,25D_3_ in bladder cancer cell migration and invasion, “wound” healing assay and Boyden chamber-based chemotactic migration or invasion assays were used. Results of the “wound” healing assay showed that 1,25D_3_ suppressed migration in 253J-BV and TCCSUP cells but not in 253J or T24 cells (Figure 3). Results in the chemotactic migration assay followed a similar trend (Figure 4A). 1,25D_3_ markedly inhibited 253J-BV cell migration and modestly suppressed migration in TCCSUP cells (Figure 4A). In contrast, migration of 253J and T24 cells was not affected by 1,25D_3_ (Figure 4A). Similar findings were observed in the invasion assay (Figure 4B). These studies consistently note that 1,25D_3_ regulates migration and invasion in bladder cancer cell lines with higher invasiveness.

### 1,25D_3_ promotes the expression of miR-101-3p and miR-126-3p in 253J-BV cells

**Figure 5:**
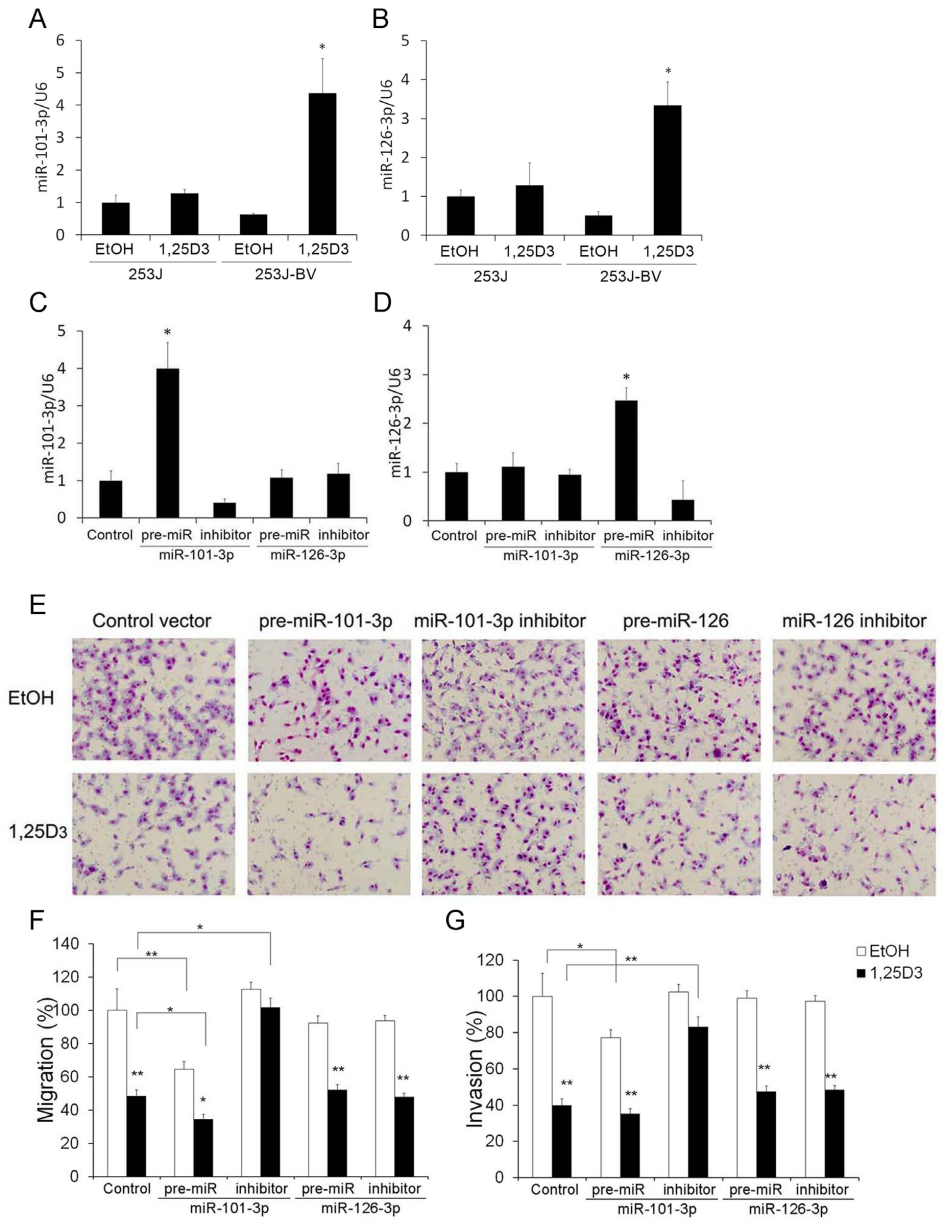
miR-101-3p is involved in 1,25D_3_ regulation of migration and invasion in 253J-BV cells **A.**-**B.** 1,25D_3_ differentially regulates miRNA expression. Cells were treated with EtOH or 1,25D_3_ for 24 h. The expression of miR-101-3p **A.** and miR-126-3p **B.** was assessed by qRT-PCR assays. *, *P* < .05; **, *P* < .01. Results are representative of three independent experiments. **C.**-**G.** 253J-BV cells were transfected with control vector, pre-miR-101-3p, pre-miR-126-3p, or the miRNA inhibitor vector for miR-101-3p or miR-126-3p for 24 h. The expression of miR-101-3p **C.** or miR-126-3p **D.** was examined by qRT-PCR. **E.** Following transfection with the above mentioned vectors, 253J-BV cells were treated with EtOH or 1,25D_3_ for 48 h and subjected to chemotactic migration or invasion assays. Representative migration images were presented. Magnification: 200x. Migration **F.** or invasion **G.** in EtOH-treated controls was normalized to 100%. *, *P* < .05; **, *P* < .01, EtOH *vs*.1,25D_3_ or as indicated.

Using miRNA PCR arrays, we found that 253J and 253J-BV cells have distinct miRNA expression profiles, which were regulated differently by 1,25D_3_ [37]. miR-101-3p and miR-126-3p were selected for further investigation because of the presence of VDREs in their promoter regions and their reported roles in migration and invasion [45, 46]. 1,25D_3_ enhanced the expression of miR-101-3p (Figure 5A) and miR-126-3p (Figure 5B) in 253J-BV but not 253J cells.

### miR-101-3p contributes to 1,25D_3_ inhibition of 253J-BV cell migration and invasion

To study the role of miR-101-3p and miR-126-3p in 1,25D_3_ inhibition of bladder cancer cell migration and invasion, the expression of miRNAs were enhanced by transfection with specific pre-miRNA vectors to miR-101-3p and miR-126-3p or suppressed by transfection with specific miRNA inhibitor vectors for 48 h in 253J-BV cells. Successful modulation of the expression levels of miR-101-3p (Figure 5C) and miR-126-3p (Figure 5D) following the transfection with pre-miRNA or miRNA inhibitor was validated by qRT-PCR. Compared with the transfection with control vectors, transfection with pre-miRNA vector increased the expression levels of miR-101-3p or miR-126-3p and miRNA inhibitor decreased the expression level of the corresponding miRNA. 253J-BV cells transfected with control vector, pre-miRNA or miRNA inhibitor were treated with 1,25D_3_ for 48 h prior to chemotactic migration and invasion assays. miR-101-3p inhibitor partially abrogated the inhibitory effect of 1,25D_3_ on 253J-BV cell migration (Figure 5E and 5F) and invasion (Figure 5G). On the other hand, transfection of pre-miR-101-3p further suppressed migration and invasion in 253J-BV cells (Figure 5E-5G). In contrast, modulation of miR-126-3p did not affect migration (Figure 5E and 5F) or invasion (Figure 5G). These findings indicate that miR-101-3p contributes to 1,25D_3_ regulation of bladder cancer cell migration and invasion.

### 1,25D_3_ regulates miR-101-3p expression *via* transcriptional regulation of miR-101-3p

**Figure 6:**
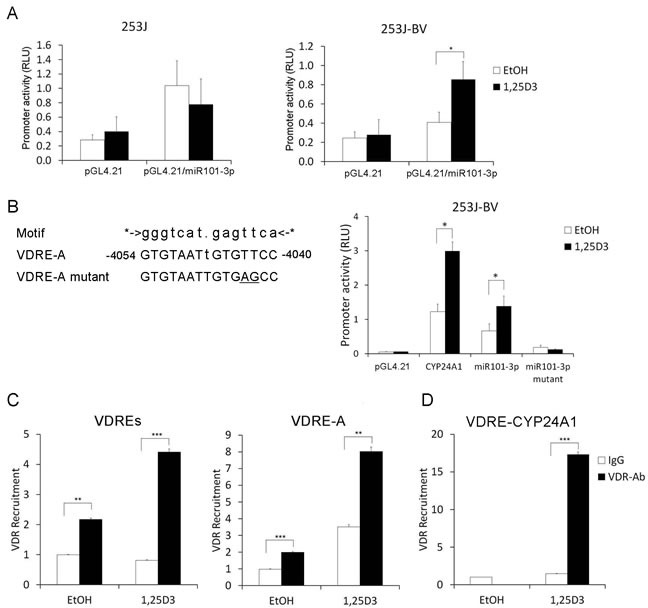
1,25D_3_ regulates miR-101-3p expression ***via*** transcriptional regulation of miR-101-3p **A.** miR-101-3p promoter construct was cloned into the promoter-less luciferase expression vector pGL4.21 immediately upstream of the luciferase gene. 253J and 253J-BV cells were seeded on 96-well plates overnight. Cells were transfected with pGL4.21 vector or pGL4.21/miR-101-3p promoter vector. The *Renilla* luciferase control construct was used as an internal control. Following 24 h of transfection, cells were treated with 1,25D_3_ at a final concentration of 500 nM for an additional 24 h. Cells were tested for their firefly and renilla luciferase activities. Data was presented as a ratio of firefly to renilla luciferase activities. Results are representative of three independent experiments. **B.** Position of putative VDRE-A in miR-101-3p promoter region was identified and shown. The sequences of putative VDRE-A and site mutated VDRE-A corresponding to consensus VDRE motif are listed. Site mutation of the predicted VDRE-A reporter was constructed (TT to AG, underlied). The wild type and mutant of miR-101-3p containing VDRE-A reporters were transiently transfected into 253J-BV cells. The induction of miR-101-3p promoter activity by 1,25D_3_ (500 nM) was measured. The *Renilla* luciferase control construct was used as an internal control. CYP24 promoter reporter construct was transfected as a positive control of 1,25D_3_ induction of promoter activity. **C.** ChIP assay was performed to pull-down VDR-DNA complex by an anti-VDR antibody from 253J-BV cells treated with 1,25D_3_. Rabbit IgG was used as an isotype control. qRT-PCR was performed with two pairs of primers amplifying DNA fragments containing potential VDREs (VDREs and VDRE-A) to detect VDR bound VDREs. **D.** Primers amplifying CYP24A1 promoter VDRE were served as positive controls. *, *P* < .05; **, *P* < .01; ***, *P* < .001.

The miR-101-3p promoter was constructed as reported previously [47]. We performed a luciferase reporter assay to test the effect of 1,25D_3_ on miR-101-3p promoter activity in 253J and 253J-BV cells. We found that 1,25D_3_ induced miR-101-3p promoter activity in 253J-BV cells but not in 253J cells (Figure 6A). To clarify the role of VDR in the regulation of the miR-101-3p gene, we searched for putative VDREs within the miR-101-3p promoter region by MAPPER: a search engine for the computational identification of putative transcription factor binding sites in multiple genomes [48]. Putative VDRE-A was identified within the miR-101-3p promoter region constructed (Figure 6B). To examine whether 1,25D_3_ regulates miR-101-3p through VDRE-A, we introduced a site mutation in VDRE-A (TT to AG, Figure 6B). CYP24 promoter reporter construct was transfected as a positive control of 1,25D_3_ induction of promoter activity (Figure 6B). Site mutation of the predicted VDRE-A diminished the induction of miR-101-3p promoter activity by 1,25D_3_ (Figure 6B), indicating that 1,25D_3_ indeed promotes miR-101-3p through VDRE-A. Further, we performed ChIP-qPCR to examine the binding between VDR and putative VDREs in chromatin associated with the miR-101-3p promoter region in 253J-BV cells. The results showed VDR was recruited to a region including VDRE-A (Figure 6C). The VDR binding to the VDRE in CYP24A1 promoter region was shown as a positive control (Figure 6D). Treatment with 1,25D_3_ increases the binding of VDR to these VDREs, suggesting that VDR can bind to VDREs in miR-101-3p promoter to induce its expression in 253J-BV cells. These results indicate that 1,25D_3_ differentially regulates miR-101-3p expression, at least partially, at the transcriptional level.

## DISCUSSION

1,25D_3_ has antitumor activity in a broad spectrum of cancer types. The major mechanisms for the antitumor effect of 1,25D_3_ are the inhibition of cancer cell proliferation, induction of apoptosis and the suppression of angiogenesis and tumor metastasis [9, 49-51]. Our previous study showed that 1,25D_3_ enhances apoptosis induced by cisplatin and gemcitabine in human bladder cancer cells T24 and UMUC3 *in vitro* and promotes the antitumor activity of cisplatin and gemcitabine in the T24 xenograft model *in vivo* [12].

1,25D_3_ has shown anti-metastatic effects in multiple studies. 1,25D_3_ suppresses migration and invasion in colon cancer cells by inhibiting DICKKOPF-4 gene, a downstream target of Wnt/β-catenin and a Wnt pathway antagonist [16]. 1,25D_3_ reduces prostate cancer cell invasion by suppressing the expression of MMP-9 and cathepsins and promoting the activity of tissue inhibitors of metalloproteinase-1 (TIMP-1) [15]. 1,25D_3_ or its analog MART-10 inhibit epithelial-mesenchymal transition (EMT) in pancreatic cancer BxPC-3 and PANC cells by down-regulating the expression of Snail, Slug and vimentin, which is accompanied by decreased migration and invasion [52]. 1,25D_3_ or its analog 22-oxa-1,25D_3_ suppresses lung metastasis of Lewis lung carcinoma (LLC) cells in an experimental metastasis model [53]. On the other hand, vitamin D deficiency promotes metastatic cancer cell growth in several metastasis models. In vitamin D-deficient mice, enhanced growth of breast cancer cells injected into the tibia and larger osteolytic lesions are observed compared to vitamin D-sufficient mice [19, 20]. Similarly, prostate cancer cell growth in bone is greater in vitamin D-deficient mice [54]. Low level of serum vitamin D levels has been associated with poorer survival for many types of cancers including lung, breast, colon, renal cancers and lymphoma [55-59]. Taken together, the preclinical observations support an anti-metastatic role for 1,25D_3_.

Despite the extensive studies in other cancer types, little is known about the role of 1,25D_3_ in migration and invasion in bladder cancer cells. Therefore, we investigated the effect of 1,25D_3_ on the metastatic potential of four bladder cancer cells. These cells have different endogenous VDR levels and in each cell VDR expression is further induced by 1,25D_3_. T24 and 253J are low-invasive bladder cancer cell lines, while 253J-BV and TCCSUP cells are highly invasive. 253J-BV cell line is a metastatic variant derived from 253J by several rounds of *in vivo* selection for highly tumorigenic and highly metastatic cells [44]. Thus, 253J and 253J-BV cell pair presents a good model system to study the regulation of migration and invasion. 1,25D_3_ treatment had no effect on the proliferation in any of these four cell lines. On the other hand, 1,25D_3_ markedly reduced migration and invasion in 253J-BV and TCCSUP cells, but not in 253J and T24 cells. These findings indicate that 1,25D_3_ has differential effect on migration and invasion in low-invasive and high-invasive bladder cancer cells. We previously showed that 1,25D_3_ inhibits SCC cell migration and invasion through the up-regulation of E-cadherin and down-regulation of MMP-2 and MMP-9 [13]. However, 1,25D_3_ did not affect the expression of E-cadherin, MMP-2 nor MMP-9 in bladder cancer cell lines (data not shown).

In certain cancer cells, 1,25D_3_ inhibits cell proliferation as well as migration and/or invasion [60, 61]. In this study, although 1,25D_3_ inhibited migration and invasion in highly invasive bladder cancer cells, it did not affect cell proliferation in any of the four bladder cancer cells. Previous reports also demonstrate similar differential impact on proliferation and migration by 1,25D_3_. In B16 melanoma cells, 1,25D_3_ did not alter cell proliferation nor influence tumor growth in the xenograft model [51]. In contrast, 1,25D_3_ inhibited B16 invasion *in vitro* and metastasis in both experimental and spontaneous metastasis model [51]. In another study, 1,25D_3_ did not affect cell growth in breast cancer cell lines SUM-149 and MDA-MB-231, which have comparable VDR levels [62]. However, 1,25D_3_ inhibited migration and invasion in the inflammatory SUM-149 cells but not the non-inflammatory MDA-MB-231 cells [62]. These findings indicate that 1,25D_3_ regulates proliferation, migration and invasion in a cell-specific manner.

miRNAs play important roles in the development, progression and metastasis of cancer. We previously showed that 1,25D_3_ differentially regulates miRNA expression in 253J and 253J-BV cells [37]. In the current study, we selected two differentially regulated miRNAs by 1,25D_3_ in 253J and 253J-BV cells, miR-101-3p and miR-126-3p, for further investigation on their potential contribution to migration and invasion. Studies using pre-miRNA or miRNA inhibitor revealed that miR-126-3p is not involved in the reduction of migration and invasion by 1,25D_3_ in 253J-BV cells. In contrast, pre-miR-101-3p further reduced, while miR-101-3p inhibitor further promoted, migration and invasion in 253J-BV cells treated with 1,25D_3_. These findings indicate that miR-101-3p contributes, at least partially, to 1,25D_3_ inhibition of metastatic potential in 253J-BV cells.

Several panels of miRNAs have been reported to be dysregulated in bladder cancer tissue samples and/or urine samples [63]. An increasing number of studies indicate that 1,25D_3_ regulates the expression of miRNAs in many cancer cell types including prostate cancer, colon cancer, ovarian cancer, lung cancer, breast cancer, melanoma and leukemia [33]. The subsequent expression change of the target gene(s) of the modulated miRNA contributes to the anti-tumor effects of 1,25D_3_ [33].

1,25D_3_ may regulate miRNA expression through a direct VDRE-mediated mechanism or an indirect mechanism which affects the genesis of mature miRNA. We identified potential VDREs in the promoter region of miR-101-3p. Luciferase reporter assay results show that 1,25D_3_ enhances the promoter activity of miR-101-3p in 253J-BV cells but not in 253J cells. Site mutation in the predicted VDRE abolished 1,25D_3_-induced miR-101-3p promoter activity, confirming the contribution of the identified VDRE. ChIP-qPCR assay confirms the recruitment of VDR to putative VDREs in miR-101-3p promoter region. These findings demonstrate that 1,25D_3_ regulates miR-101-3p expression, at least in part, at the transcriptional level. In the meantime, participation of non-transcriptional mechanism cannot be excluded. Previous studies also documented VDRE-dependent regulation of miRNA expression by 1,25D_3_. For instance, 1,25D_3_ induces the synthesis of miR-498 through a functional VDRE located in the 5′ regulatory region, which leads to the down-regulation of telomerase and inhibition of ovarian cancer cell proliferation [34]. In osteoblasts, 1,25D_3_ induces the expression of miR-637 and miR-1228 by two distinct mechanisms [64]. The induction of miR-1228 by 1,25D_3_ is through VDRE-mediated transactivation of a host gene LRP1; while the regulation of miR-637 is by intronic VDRE-mediated induction without the transactivation of the host gene DAPK3 [64].

miR-101 has been reported to be involved in the progression of several cancer types. The regulation of miR-101-3p may be through multiple mechanisms. miR-101 expression is reduced in various cancer tissues and cell lines such as breast cancer, gastric cancer, and intrahepatic cholangiocarcinoma (ICC) [65-67]. miR-101 is also down-regulated in bladder cancer tissue samples [68]. Overexpression of miR-101 inhibits the proliferation and invasion of bladder cancer cell line T24, potentially through the regulation of c-FOS expression [69]. In breast cancer cells, miR-101 promotes apoptosis and inhibits cell proliferation, which is associated with increased expression of EYA1 [65]. miR-101 suppresses migration and invasion in ICC cells through targeting VEGF-C, at least in part [67]. Cyclooxygenase-2 is another target of miR-101 that has been shown to contribute to enhanced sensitivity of bladder cancer cells to cisplatin [70]. LncRNA SPRY4-IT1 directly interacts with and inhibits miR-101-3p expression, leading to EZH2 upregulation and enhanced proliferation and metastasis in bladder cancer T24T cells [71]. These studies support the contribution of miR-101 in cancer development and metastasis.

In conclusion, we show that 1,25D_3_ inhibits cell motility and invasion in the metastatic human bladder cancer cell lines 253J-BV and TCCSUP. In contrast, 1,25D_3_ treatment has no effect on migration and invasion in low invasive cell lines 253J and T24. The suppression of migration and invasion is partially through the transcriptional induction of miR-101-3p by 1,25D_3_. Further investigation is needed to elucidate the mechanism for 1,25D_3_-mediated differential regulation of migration and invasion in bladder cancer cells. However, the observed anti-metastatic activity of 1,25D_3_ in multiple preclinical model systems supports its evaluation in the clinical setting.

## MATERIALS AND METHODS

### Materials

1,25D_3_ (Hoffmann-LaRoche, Nutley, NJ) was reconstituted in 100% ethanol (EtOH) and stored, protected from light, under nitrogen gas at −80°C. Anti-vitamin D receptor (VDR) antibodies (D-6, used in the immunoblot analysis and sc-1008 X, used in ChIP assay) were purchased from Santa Cruz Biotechnology (Santa Cruz, CA). Anti-actin (CP-01) was from Calbiochem (San Diego, CA).

### Cell culture

Four human bladder cell lines were used: T24, 253J, 253J-BV and TCC-SUP. T24, 253J and TCCSUP cells were purchased from ATCC (Manassas, VA) and passaged within 6 months after the receipt or resuscitation of the frozen cells. Cell lines were authenticated by ATCC with short tandem repeat (STR) DNA profiling and cytogenetic analysis.

253J-BV cell line is a metastatic variant derived from 253J and was generously provided by Dr. Ashish Kamat (MD Anderson Cancer Center) which was characterized previously [44, 72]. 253J cells were maintained in RPMI 1640 supplemented with 10% fetal bovine serum (FBS), penicillin and streptomycin. 253J-BV cells were maintained in modified Eagle's MEM supplemented with 10% FBS, vitamins, sodium pyruvate, L-glutamine, penicillin, streptomycin, and nonessential amino acids. T24 was cultured in McCoy's 5A media supplemented with 10% FBS, penicillin and streptomycin. TCCSUP was cultured in DMEM media supplemented with 10% FBS, penicillin and streptomycin.

### MTT cell proliferation assay

253J, 253J-BV, TCCSUP and T24 cells were plated in 96-well tissue culture plates. Cells were treated with EtOH or varying concentrations (0 - 1000 nM) of 1,25D_3_ for 24-72 h. Cell growth was assessed by MTT assay as previously described [73].

### “Wound” healing assay

A confluent monolayer of 253J, 253J-BV, TCCSUP or T24 cells was cultured overnight and a scratch was introduced with a pipette tip and images of cell migration into the wound were captured at 0, 24 and 48 h using a light microscope.

### Chemotaxis migration assay

Chemotactic migration activity was measured by Boyden-chamber assay using BD BioCoat Control Inserts. 253J, 253J-BV, T24 and TCCSUP cells were plated in insert chambers in serum-free media that they are maintained with. The lower chambers were filled with media supplemented with 5% FBS. After 16 h of incubation, cells that did not migrate were removed from the upper chambers with a cotton swab, and cells that migrated through the pore membrane were identified by Diff-Quik^®^ Stain Set (Dade Behring, Newark, DE), examined and counted under a bright field microscopy.

### Invasion assay

The invasion activity was measured by Boyden-chamber assay using BD BioCoat Matrigel Invasion Chambers as in the chemotaxis migration assay except for a longer incubation time of 48 h. The results are expressed as follows: % invasion index = (the number of cells migrating through the collagen-coated membrane/the number of cells migrating through the uncoated control membrane) × 100.

### Immunoblot analysis

Human bladder cancer cells 253J, 253J-BV, T24 and TCCSUP were treated with vehicle control EtOH or 500 nM 1,25D_3_ for 48 h. Cells were harvested and lysates prepared as previously described [74]. Immunoblot analysis was performed as described [74].

### miRNA qRT-PCR assays

Human bladder cancer cells 253J and 253J-BV were treated with EtOH or 500 nM 1,25D_3_ for 48 h. RNA including small RNAs was isolated with miRNeasy Mini kit following the manufacturer's instructions (Qiagen, Valencia, CA). cDNA was synthesized with 100 ng RNA using All-in-One™ miRNA First-Strand cDNA Synthesis Kit Genecopoeia (AMRT-0020, Rockville, MD) following the manufacturer's protocol. The qPCR primers used are: hsa-miR-101-3p (HmiRQP0021), hsa-miR-126-3p (HmiRQP0099) snRNA U6 (HmiRQP9001). U6 was used as an internal control. qPCR was performed in triplication with the All-in-One™ qPCR Mix (Genocopoeia, AOPR-1000) on a 7300 Real-Time PCR System with the standard protocol (Applied Biosystems, Carlsbad, California).

### Transfection

For miRNA over-expression studies, 253J or 253J-BV cells were transiently transfected with 1000 ng of precursor miRNA expression vector for hsa-mir-101-1 (HmiR0009-MR04-B), hsa-mir-126 (HmiR0153-MR04-B) or control vector (CmiR0001-MR04, all from Genecopoeia) for 48 h. To inhibit miRNA expression, 253J or 253J-BV cells were transiently transfected with 1000 ng of miRNA inhibitor vector against hsa-miR-101-3p (HmiR-AN0021-AM01), hsa-miR-126-3p (HmiR-AN0099-AM01) or control vector (pEZX-MR04, Genecopoeia) for 48 h. All transient transfection was performed in triplicate with the transfection reagent DharmaFECT1 (Dharmacon, Lafayette, CO) according to the manufacturer's protocol. The effect of transfection on the expression levels of miR-101-3p and miR-126-3p was evaluated by qRT-PCR as described above.

### Luciferase reporter assay

The fragment of the miR-101-3p promoter region, as described by Sheng Y [47], was amplified by PCR from human genomic DNA using primers NheI-miR-101-3p-P (5′-CTAGCTAGCGAACCTGCAGGGAAGTGGAGT-3′) and XhoI-miR-101-3p-R (5′- GCAGCTCGAGGGTTGGAGACGTGAGGAGGC-3′) with PfuUltra high-fidelity DNA polymerase (Stratagene, San Diego, CA). The NheI- and Xho-digested amplicon was cloned into the promoter-less luciferase expression vector pGL4.21 (Promega, Madison, WI) immediately upstream of the luciferase gene to produce the plasmid pGL4.21/miR101-3p. 253J and 253J-BV cells were transfected with 100 ng of the pGL4.21/miR101-3p or pGL4.21 constructs along with 20 ng of a renilla luciferase control construct (Promega). All transfections were carried out in triplicate wells of 96-well plates for 24 h. Following transfection, cells were treated with 1,25D_3_ (500 nM) for an additional 24 h and harvested. Firefly and renilla luciferase activities were measured using the Dual-Luciferase Reporter Assay System (Promega) according to the manufacturer's instructions.

### Site mutation and luciferase reporter assay

To generate site mutations in the predicted VDRE of miR101-3p promoter luciferase reporter, TT dinucleotides in VDRE were replaced with AG using PCR-based site-directed mutagenesis (Q5 site-directed mutagenesis kit, New England bioLabs, Beverly, MA)(Figure 6B). The forward (5- gtgagccTGGAACAGCCCAAGGCTG-3) and reverse (5- aattacacTCCACTTCCCTGCAGGTTC-3) primers were designed according to the manufacturer's instructions. The PCR reactions were started with incubation at 98°C for 30 sec followed by 25 cycles of 10 s at 98°C, 20 s at 68°C, and 2 min at 72°C and a final 2-min extension at 72°C. The PCR products were treated with kinase, ligase and DpnI enzyme mixture according to the manufacturer's instructions. After transformation, colonies were screened and the sequences of the mutant plasmids were confirmed by DNA sequencing. 253J-BV cells were transfected with the pGL4.21/miR101-3p or its mutant pGL4.21 constructs along with a Renilla luciferase control construct (Promega). All transfections were carried out in triplicate wells of 96-well plates. Twenty-four hours after transfection, cells were treated with 1,25D_3_ (500 nM) for an additional 24 h and harvested, and firefly and Renilla luciferase activities were measured using the Dual-Luciferase Reporter Assay System (Promega) according to the manufacturer's instructions.

### Quantitative chromatin immunoprecipitation-PCR (ChIP-qPCR)

ChIP-qPCR was performed as previously described [75]. 253J-BV cells (3×10^6^) were plated in 100 mm-dishes overnight and then treated with 500 nM of 1,25D_3_ or EtOH for 1 h. Formaldehyde was added to cross-link proteins to DNA by adding drop-wise directly to the media for a final concentration of 1% and incubated at 37°C for 10 min. Glycine was added to a final concentration of 125 mM to the media and incubated 37°C for 5 min. Cells were harvested by scraping into ice-cold PBS supplemented with a protease inhibitor cocktail (Roche, Mannheim, Germany) and washed with cold PBS twice. After centrifugation, cell pellets were re-suspended in 0.5 ml of lysis buffer on ice for 10 min. The lysates were sonicated to shear DNA to an average fragment size of 300-1000 bp in length. Cellular debris was removed by centrifugation. 5% of lysate was collected as DNA input. Lysates were diluted 1:10 (v/v) in ChIP dilution buffer (0.01% SDS, 1.1% Triton X-100, 1.2mM EDTA, 16.7 mM Tis-HCl, pH 8.1, 167 mM NaCl) and incubated with a salmon sperm DNA/protein A/agarose slurry (Upstate Biotechnology, Lake Placid, NY) at 4°C for 1 h with agitation. Chromatin suspension was incubated with VDR antibody (12 μg) or IgG control, and protein A/G beads overnight. The beads were pelleted by centrifugation at 4°C and washed with a series of buffers: low salt immune complex washing buffer (0.1% SDS, 1% Triton X-100, 2mM EDTA, 20 mM Tis-HCl, pH 8.1, 150 mM NaCl), high salt immune complex washing buffer (0.1% SDS, 1% Triton X-100, 2mM EDTA, 20 mM Tis-HCl, pH 8.1, 500 mM NaCl), LiCl washing buffer (0.25 M LiCl, 1% sodium deoxycholate, 10 mM Tris-HCl pH 8,1 1% NP-40, 1 mM EDTA) and TE buffer. DNA was eluted twice from protein A/G beads with elution buffer (1% SDS, 0.1 M NaHCO_3_) rotated for 15 min at room temperature. The cross-linking was reversed by adding 5M NaCl to a final concentration of 200 mM and incubated at 65°C overnight. The remaining proteins were digested by adding proteinase K and incubated at 45°C for 1 h. The DNA was purified by phenol/chloroform/isoamyl alcohol (25/24/1) and precipitated with 0.1 volume of 3 M sodium acetate (pH 5.2) and two volumes of EtOH using glycogen as a carrier. A fragment encompassing the putative vitamin D response element (VDRE) on miR-101-3p promoter region was amplified by qPCR and normalized to input DNA by the following specific primer pairs: VDREs (including VDRE-A and an additional VDRE): (5′-CTGCAGGGAAGTGGAGTGTAAT-3′ and 5′-TATATGGGCCTCTCCCCTTCAA-3′), VDRE-A (5′-CTGCAGGGAAGTGGAGTGTAAT-3′ and 5′-AATGGACAGCTAAAGGAGCCAA-3′). The VDRE in CYP24A1 promoter region was used as a positive control using the following primers: huCYP24-F-292 5′-AGCACACCCGGTGAACTC-3′and

huCYP24-R-152 5′-TGGAAGGAGGATGGAGTCAG-3′.

### Statistical analyses

The differences between control and treatment groups were analyzed for statistical significance using the two-tailed student's *t*-test.

## Abbreviations

EMT: epithelial mesenchymal transition
EtOH: ethanol
FBS: fetal bovine serum
ICC: intrahepatic cholangiocarcinoma
SCC: squamous cell carcinoma
TIMP-1: tissue inhibitors of metalloproteinase-1
VDR: vitamin D receptor
VDRE: vitamin D response element
